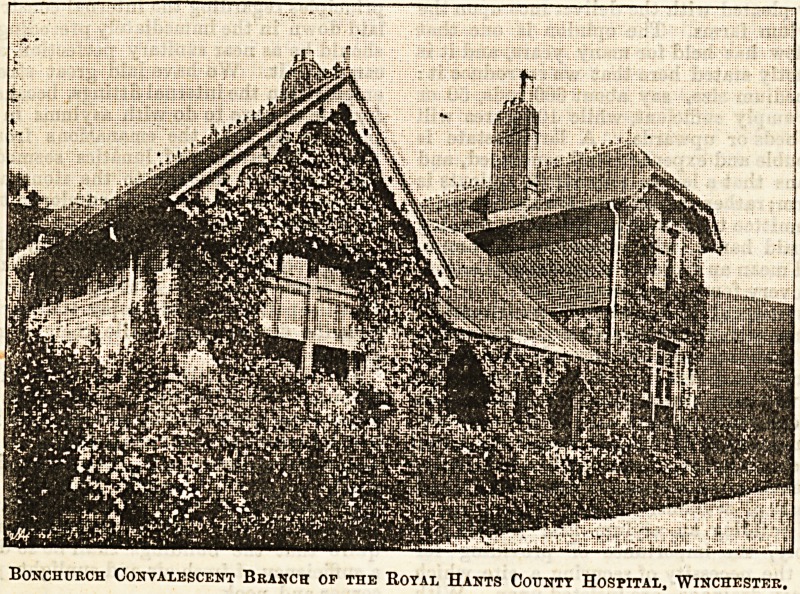# The Convalescent Home at Bonchurch

**Published:** 1892-07-30

**Authors:** 


### WITHIN THE HOSPITALS.

### VII.—THE CONVALESCENT HOME AT BONCHURCH.

This institution is in connection with the Royal Hants County Hospital, and it will be seen from the accompanying illustration that it presents a most attractive appearance. During the year 1891 eighty patients were admitted, forty from the Royal Hants County Hospital, in addition to forty private patients. The Home excites much interest among the residents at Bonchurch, and the Hon. Miss Scott sets an excellent example by granting the patients the privilege of using her garden during the summer months. Several other residents take a great interest in the children, providing them with toys and books, and sometimes even with clothes. The Home is utilised by the Committee for the reception of such members of their nursing staff as may need a change of air and rest, an example which other institutions might usefully follow. The payment of 4s. per week is made by, or on behalf of, each patient sent from the hospital, except in the case of domestic servants in service, who are charged 10s. 6d. per week, but in cases of exceptional urgency or poverty patients are admitted free. Each patient remains, on an average, about four weeks in the Home, but no case of infectious or contagious disease, consumption or epilepsy, nor patients suffering from incurable or mental disease, are admitted. The Home affords a delightful residence for the patients, is in charge of a Matron, and all the arrangements seem to be satisfactory. The annual expenditure amounts to about £300, towards which the patients contributed £140 in the year ended March 31st, 1891. The balance has been made up by subscriptions and donations especially given to the Convalescent Home Branch of the Royal Hants County Hospital.

Bonchurch Convalescent Branch of the Royal Hants County Hospital, Winchester.

## Figures and Tables

**Figure f1:**